# At-will chromatic dispersion by prescribing light trajectories with cascaded metasurfaces

**DOI:** 10.1038/s41377-020-0335-7

**Published:** 2020-05-27

**Authors:** Andrew McClung, Mahdad Mansouree, Amir Arbabi

**Affiliations:** Department of Electrical and Computer Engineering, University of Massachusetts Amherst, 151 Holdsworth Way, Amherst, MA 01003 USA

**Keywords:** Metamaterials, Nanophotonics and plasmonics, Sub-wavelength optics, Metamaterials, Nanophotonics and plasmonics

## Abstract

Chromatic dispersion spatially separates white light into colours, producing rainbows and similar effects. Detrimental to imaging but essential to spectroscopy, chromatic dispersion is the result of material properties in refractive optics and is considered an inherent characteristic of diffractive devices such as gratings and flat lenses. Here, we present a fundamental relation connecting an optical system’s dispersion to the trajectories light takes through it and show that arbitrary control over dispersion may be achieved by prescribing specific trajectories, even in diffractive systems. Using cascaded metasurfaces (2D arrays of sub-micron scatterers) to direct light along predetermined trajectories, we present an achromatic twisted metalens and experimentally demonstrate beam deflectors with arbitrary dispersion. This new insight and design approach usher in a new class of optical systems with wide-ranging applications.

## Optics: A new trajectory for controlling chromatic dispersion

A new technique that uses two-dimensional (2D) metasurfaces to control the dispersion of light in optical systems could pave the way for new devices for a wide range of optical applications. Chromatic dispersion occurs when the different wavelengths of a light beam arrive at their destination at different times. Although useful in applications like spectroscopy, chromatic dispersion must be compensated for in wideband imaging systems. Now, a team of researchers led by Amir Arbabi from the University of Massachusetts Amherst in the United States has developed a new technique that uses 2D arrays of sub-micron scale metasurfaces that shape the optical wavefronts of light with subwavelength resolution. By directing the light along predetermined trajectories, the technique allows chromatic dispersion to be controlled and could be used in applications from photography and astronomy to microscopy.

## Introduction

Chromatic dispersion has a long history in optical science. Newton’s seminal discovery that white light is formed of constituent colours^1^ relied on triangular glass prisms, which, like other refractive elements, are dispersive due to their wavelength-dependent refractive indices^2^. In contrast, diffraction gratings and flat lenses exhibit a strong dispersion that is not material dependent but is instead considered inherent to the diffraction phenomenon^3^. Different chromatic dispersions are desirable in different optical systems. Wideband imaging systems used in photography, astronomy, and microscopy should be achromatic because chromatic dispersion blurs images and is regarded as an aberration. On the other hand, optical systems such as spectrometers benefit from strong chromatic dispersion. The traditional approach for engineering chromatic dispersion is based on cascading refractive elements made of different materials^2^ or pairing refractive and diffractive elements^4^.

The advent of highly efficient metasurfaces has spurred significant renewed interest in engineering chromatic dispersion^5–14^. Optical metasurfaces are two-dimensional (2D) arrays of scatterers (or meta-atoms) that shape optical wavefronts with subwavelength resolution. They can replace conventional elements^15–20^, facilitate the implementation of novel functionalities by offering unprecedented control over the flow of light^21–25^, and enable planar optical systems that can be mass produced similarly to semiconductor chips^26–29^. Like diffractive elements, metasurfaces have wavelength-independent phase profiles due to phase wrapping^7^, and the conventional approach of chromatic correction through cascading has been proven ineffective in focusing systems made only of such elements^30^. New approaches that permit operation at a few discrete wavelengths have been proposed^6,7,31^, but such approaches are not viable in wideband applications requiring correction over a continuous spectral range (i.e., achromatic components). Consequently, achromatic systems employing metasurfaces have been limited to diffractive-refractive hybrids^9,32^ or small metasurfaces that exploit meta-atom dispersion^8,11–14^. Metasurfaces function as correctors in hybrid diffractive-refractive systems, reducing the overall size of the system, but the refractive components are the main focusing elements in such systems. Metasurfaces relying on meta-atom dispersion to correct chromatic aberrations offer the desired planar form factor, but their size and numerical aperture are inherently limited by the highest meta-atom quality factors that can reliably be attained^8^.

Starting from first principles, we present a fundamental relation between the ray trajectories in optical systems and their chromatic response. Contrary to the prevalent description of chromatic dispersion that attributes it to optical elements such as gratings or metalenses, the new relation reveals variations in ray trajectories as the origin of chromatic dispersion. This relation offers clear insight into the chromatic response of optical systems and establishes a framework for designing unconventional optical systems with arbitrary chromatic dispersions. Specifically, we show that an optical system is achromatic if all ray trajectories have equal optical group lengths (OGLs). Therefore, the design of achromatic systems involves selecting a set of ray trajectories with equal OGLs and then designing components (e.g., metasurfaces) to direct light along those trajectories.

## Results

**Fig. 1:**
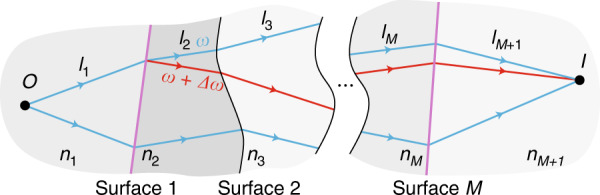
Illustration of focusing by a cascaded metasurface system. Rays of angular frequency *ω* (blue lines) travel from object point *O* through a system of *M* surfaces to image point *I*. The system is achromatic if rays of a different angular frequency (red lines) leaving *O* also converge at *I*. Purple interfaces represent metasurfaces, and black interfaces represent boundaries between materials of different refractive indices.

We begin by describing the achromatic condition and then discuss its extension to arbitrary chromatic dispersions. Consider the optical system shown in Fig. 1, which is constructed of different transparent media. The surfaces separating the media may be non-planar and may include a metasurface with a phase profile *φ*_*m*_. Suppose that the system is designed to bring a set of rays of angular frequency *ω* originating at an object point *O* into focus at an image point *I* (as shown in Fig. 1). The total phase accumulated by a fiducial ray in this set can be expressed as

1 $$\Phi = \mathop {\sum }\limits_{m = 1}^{M + 1} \frac{\omega }{c}n_ml_m + \mathop {\sum }\limits_{m = 1}^M \varphi _m$$

where *c* is the speed of light in vacuum, *l*_*m*_ is the path length between surfaces *m*−1 and *m* inside a material with refractive index *n*_*m*_, and *φ*_*m*_ is the phase imparted by the *m*th surface. If the *m*th surface does not include a metasurface, then *φ*_*m*_ = 0. Because the system focuses the rays at *I*, all rays’ paths acquire the same Φ (ref. ^2^).

If the frequency is changed to $\omega + \Delta \omega$, the fiducial ray will acquire a different total phase of $\Phi + \Delta \Phi$. We will designate as ‘achromatic’ a system in which the entire set of rays over a continuous frequency range near *ω*, to the first-order approximation in $\Delta \omega$, is focused to *I*. The phase change $\Delta \Phi$ can be partitioned into two contributions. First, the frequency change will cause the ray to acquire a different total phase along its original trajectory. Second, each surface will deflect the ray by a different angle, altering its trajectory. The contribution of this second term is zero (see the Supplementary Information), so the system is achromatic if and only if $\Delta \Phi = l_{\mathrm g}\Delta \omega /c$ is the same for all rays, where

2 $$l_{\mathrm g} = \mathop {\sum }\limits_{m = 1}^{M + 1} n_{m_{\mathrm g}}l_m + \mathop {\sum }\limits_{m = 1}^M c\varphi {\prime}_m$$

Here, $n_{m_{\mathrm g}} = {\mathrm d}(\omega n_m)/{\mathrm d}\omega$ represents the group index for the *m*th material, and $\varphi {\prime}_m = \partial \varphi _m/\partial \omega$ is the dispersion of the phase imparted by the *m*th surface. The travel time for a narrowband pulse along the ray’s trajectory is the group delay $\tau _{\mathrm g} = \Delta \Phi /\Delta \omega$; hence, we refer to $l_{\mathrm g} = c\tau_{\mathrm g}$ as the OGL. In the following, we will describe surfaces with frequency-dependent phase profiles $\left( {\varphi {\prime} \ne 0} \right)$ as ‘dispersive’ and those with frequency-independent profiles $\left( {\varphi {\prime} = 0} \right)$ as ‘non-dispersive.’ Although their phase profiles do not change with frequency, non-dispersive surfaces do disperse light (consider, e.g., a diffraction grating).

The group delay imposed by a single-layer dispersive metasurface $\left( {\varphi{\prime} \ne 0} \right)$ has recently been engineered to modify the chromatic response of high-contrast transmitarray^8^ and Pancharatnam–Berry^11–14^ metasurfaces. Because the delay is proportional to the quality factors of the meta-atoms^8^, the achievable group delays are limited, restricting the applicability of this approach for chromatic correction to narrow bandwidths, very small metasurfaces, or both. However, dispersion can also be controlled using the first term in Eq. (2), which is related to the geometric lengths over which a ray travels between surfaces. In systems of non-dispersive metasurfaces $\left( {\varphi {\prime} = 0} \right)$, an achromatic response is achieved by imposing the requirement of a constant $l_{\mathrm g} = \mathop {\sum }\nolimits_{m = 1}^{M + 1} n_{m_{\mathrm{g}}}l_{m}$ for all rays. Therefore, an achromatic optical system can be designed by first identifying a set of ray paths that satisfy the equal-OGL condition and then directing the rays along these paths using metasurfaces. We illustrate this novel approach by discussing the design of an achromatic beam deflector and an achromatic metalens doublet.

**Fig. 2:**
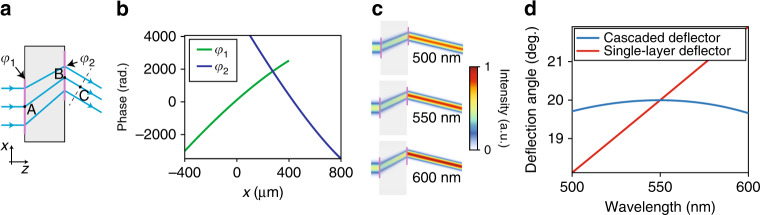
Achromatic beam deflector. **a** Schematic of an achromatic bilayer metasurface beam deflector. **b** Phase profiles of the metasurfaces composing the beam deflector shown in **a**. **c** Full-wave simulation results for the deflection of a Gaussian beam by the beam deflector. **d** Wavelength dependence of the deflection angle for the achromatic bilayer beam deflector and a single-layer metasurface beam deflector.

Beam deflectors are the basic building blocks of gradient metasurfaces, and such metasurfaces can be considered beam deflectors with spatially varying deflection angles. A single-layer, non-dispersive metasurface beam deflector that deflects normally incident light by an angle *θ* exhibits a grating dispersion of ${\mathrm d}\theta /{\mathrm d}\lambda = {\mathrm{tan}}\theta /\lambda$ (ref. ^2^). As shown in Fig. 2a, a metasurface beam deflector can be realized using two parallel non-dispersive $\left( {\varphi {\prime} = 0} \right)$ metasurfaces by choosing points *O* and *I* far from the metasurfaces along the normal direction and at an angle *θ* with respect to the normal direction, respectively. To achieve achromatic beam deflection (i.e., ${\mathrm d}\theta /{\mathrm d}\lambda = 0$), the two metasurfaces should be designed such that the OGL from *O* to *I*, or equivalently (as shown in Fig. 2a) $l_{\mathrm g} = n_{\mathrm g}l_{AB} + l_{BC}$, is the same for all ray paths (*n*_g_: group index of the material separating the metasurfaces). Constraining *l*_g_ to a fixed value determines the path taken by a ray incident at any point *A* on the first metasurface. Rays incident at different points on this surface must be deflected by different angles to meet the imposed constraint. Using the deflection angles of these paths and the grating equation, the phase profiles are obtained (see the Supplementary Information). Figure 2b shows the phase profiles for a 20° bilayer metasurface beam deflector produced via this procedure. Simulated intensity distributions depicting the deflection of Gaussian beams with different wavelengths are shown in Fig. 2c, and the wavelength dependence of the deflection angles for the bilayer and single-layer beam deflectors is shown in Fig. 2d, demonstrating the achromatic response of the bilayer deflector at its design wavelength of 550 nm.

Now, we consider the achromatic on-axis focusing of a normally incident beam by cascaded non-dispersive metasurfaces. According to the equal-OGL condition, if all of the metasurfaces have paraxial regions, then a set of cascaded parallel metasurfaces cannot be achromatic because the axial ray has the minimum OGL. However, an achromatic cascaded metalens with an annular aperture can be realized if the metasurfaces are designed to deflect the rays along paths of equal OGLs. Unlike the group delay approach described above, annular apertures impose no restrictions on the lens size and hence enable a larger space–bandwidth product^33,34^, the ultimate determinant of the information content of an image.

**Fig. 3:**
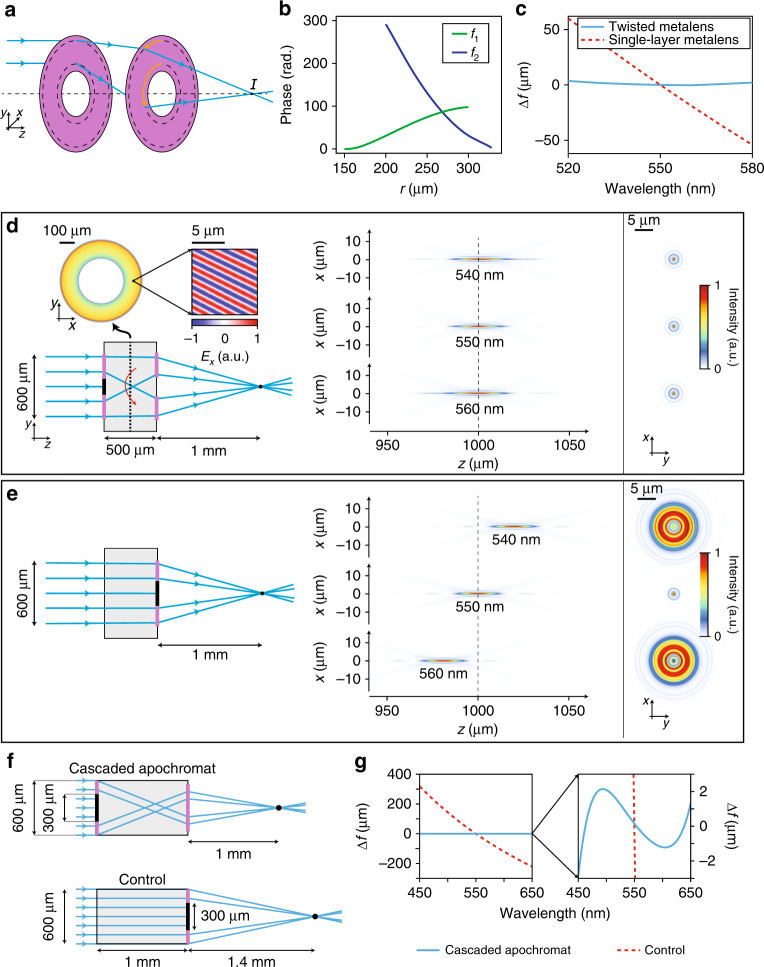
Achromatic and apochromatic bilayer metalenses. **a** Illustration of a twisted achromatic bilayer metalens. **b** Radial portions of the phase profiles of the two metasurfaces of the metalens shown in **a**. **c** Wavelength dependence of the focal lengths of the twisted bilayer metalens shown in **a** and a single-layer metalens. **d** Ray schematic, intensity, and field distribution on a plane between the metasurfaces of the twisted metalens (left) and the axial-plane (centre) and focal-plane (right) intensity distributions at three different wavelengths. **e** Ray schematic (left) and axial-plane (centre) and focal-plane (right) intensity distributions for an annular single-layer metalens. **f** Illustration of an apochromatic bilayer metalens and an annular single-layer metalens with the same aperture and NA, which serves as a control. **g** Wavelength dependence of the focal lengths of the metalenses shown in **f**. The apochromatic bilayer metalens satisfies the achromaticity criterion at two wavelengths, *λ* = 494.1 nm and *λ* = 604.5 nm.

Figure 3a shows an example of a bilayer metalens that achieves equal OGLs by deflecting the rays to different angles along the azimuthal direction. Incident rays closer to the optical axis are deflected by larger azimuthal angles by the first metasurface to compensate for the shorter length they travel between the second metasurface and the focal point *I*. As a proof of concept, we have designed a circularly symmetric bilayer metalens with an annular aperture (150 μm < *r*_1_ < 300 μm), selecting $\varphi _1 = N\theta + f_1\left( r \right)$ and $\varphi _2 = - N\theta + f_2\left( r \right) + \omega /c\sqrt {r^2 + f^2}$ for the two metasurfaces, which are separated by a 500-μm-thick substrate with a refractive index of 1.46. We chose *N* = 750 and a focal length of *f* = 1 mm; $f_1(r)$ and $f_2(r)$ were obtained by setting *l*_g_ = 1785 μm for all ray paths and are shown in Fig. 3b. Two-dimensional phase profiles for the metasurfaces are presented in Supplementary Fig. S1.

In the wave optics picture, the first metasurface twists the incident light, generating a helical beam with a large (*N* = 750) orbital angular momentum, and the second metasurface removes the wavefront’s helicity and focuses the beam to the point *I*. Different radial portions of the finely structured helical beam traverse the substrate at different group velocities via an effect similar to one that has been recently observed^35^. Portions of the wave closer to the optical axis have slower axial group velocities. A similar group velocity dependence could be achieved if the substrate had a radially dependent refractive index; thus, the combination of the homogeneous substrate and the two metasurfaces exhibits behaviour similar to that of a graded index lens.

Simulated focal length shifts for the bilayer twisted metalens and a single-layer metalens are shown in Fig. 3c. As expected, the twisted metalens is achromatic (i.e., ${\mathrm d}f/{\mathrm d}\lambda = 0$) at its design wavelength. To verify that the achromatic response is not merely due to the annular aperture, a single-layer metalens with the same annular aperture was also designed for comparison. The focal-plane intensity distributions of the twisted and annular single-layer metalenses are shown in Fig. 3d, e. Intensity distributions for the twisted metalens over a wider range of wavelengths and corresponding distributions for a single-layer metalens with a circular aperture are presented in Supplementary Fig. S2.

The twisted metalens serves as an illustration of the unconventional elements enabled by our approach, but better-performing designs are possible. To demonstrate, we have also designed a bilayer apochromatic metalens with the same aperture and focal length as the twisted metalens. This apochromat, shown in Fig. 3f, uses only radial deflections, producing a rotationally symmetric design. Similar to the twisted metalens, the apochromat was designed by selecting ray paths with equal OGLs. The operational principle can be qualitatively understood by considering the paths of two rays: The ray that enters the system farthest from the optical axis experiences the largest deflection, thus achieving the longest OGL within the substrate; this ray exits the system closest to the optical axis, producing the shortest OGL in the image space. Conversely, the ray entering the system nearest to the optical axis experiences the smallest deflection, and exits the system farthest from the optical axis, achieving the shortest OGL within the substrate and the longest one outside it. The achromaticity criterion is satisfied when the total OGLs of these two ray paths are equal. This occurs for two different OGL values ($l_{\mathrm g}^{(1)} = 2581.9\,\upmu {\mathrm{m}}$ and $l_{\mathrm g}^{(2)} = 2628.6\,\upmu {\mathrm{m}}$) at two different wavelengths (*λ*^(1)^ = 494.1 nm and *λ*^(2)^ = 604.5 nm), resulting in two stationary points in the focal length dispersion (Fig. 3g); at nearby wavelengths, the criterion is also approximately satisfied, resulting in an operational bandwidth of 200 nm. A detailed discussion of the design of the bilayer apochromat is presented in Supplementary Fig. S3 and the Supplementary Information.

Moreover, although the twisted metalens performs well for fields at normal incidence, aberrations are apparent at angles of incidence as small as 0.2° (see Supplementary Fig. S4). While these aberrations are problematic for imaging, the twisted metalens could, for example, be used as a broadband collimator or focuser in applications for which a wide field of view is unnecessary. The correction of monochromatic aberrations has previously been demonstrated^26^, and achromatic metalenses with large fields of view may be possible in systems with three or more layers.

The simple relation between the change in the accumulated phase and the OGL (i.e., $\Delta\Phi = l_{\mathrm g}\Delta \omega /c$) allows dispersion to be engineered by imposing different constraints on the OGL, thereby producing metasystems that exhibit dispersive behaviour other than achromatic dispersion or simple grating dispersion. When the frequency changes by $\Delta \omega$, the phase at each point on the wavefront changes by $\Delta \Phi = l_{\mathrm g}\Delta \omega /c$. If the OGLs of rays travelling from *O* to different points on the wavefront are all the same, then the phase changes at all points are equal: the wavefront is unchanged, and the system is achromatic. On the other hand, the variation of the wavefront with the wavelength can be engineered as desired by selecting ray trajectories that have prescribed OGLs and then designing metasurfaces to direct rays along these trajectories.

**Fig. 4:**
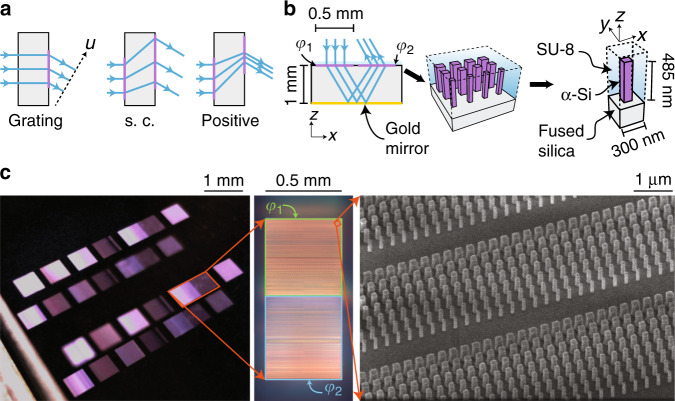
Beam deflector schematics and images. **a** Comparison of a single-layer metasurface grating beam deflector with bilayer superchromatic (s.c.) and positive-dispersion designs. **b** Schematics showing (left) the cascaded metasystem, which uses a gold mirror on the back of the fused silica substrate to ‘fold’ the cascaded systems depicted in **a**; a portion of one of the metasurfaces (centre); and a unit cell (right) showing a single meta-atom. **c** A photograph (left) showing the beam deflector patterns and their reflections, an optical microscope image (centre) of the folded achromatic beam deflector and an SEM image (right) of the nano-posts.

We illustrate this idea in Fig. 4a, which depicts an ordinary grating beam deflector alongside two beam deflectors with engineered dispersion: a superchromatic (exceeding grating dispersion) beam deflector and a beam deflector exhibiting positive dispersion. In the grating beam deflector, the OGL from *O* to the wavefront, represented by the dashed black line, increases linearly with increasing distance *u* along the wavefront, i.e., the upper ray has a larger OGL than the lower ray. With a change in frequency, different points along the wavefront acquire phase shifts that are proportional to their OGLs and linear in *u*, resulting in a wavefront tilt. The magnitude and direction of this tilt can be controlled by modifying the difference in the OGLs for different points along the wavefront. Extending and shortening the paths of the upper and lower rays, respectively, increases their OGL difference, thereby increasing the rate of wavefront tilt with respect to frequency, producing a superchromatic beam deflector. This is depicted schematically in the superchromatic design in Fig. 4a. By contrast, shortening the upper ray’s path and extending that of the lower ray to the point that the OGL of the lower ray exceeds that of the upper ray, as shown in the positive-dispersion design in Fig. 4a, inverts the direction of the wavefront tilt. Such a beam deflector will exhibit positive dispersion, similar to refractive prisms made of materials with normal dispersion.

To demonstrate the concept and design methodology, we designed, fabricated, and characterized achromatic, superchromatic, and positive-dispersion beam deflectors that have 0, 3, and −1 times the dispersion, respectively, of an ordinary grating. The beam deflectors are composed of two cascaded metasurfaces (as shown in Figs. 2a and 4a) and are designed to deflect a normally incident 850-nm beam to an angle of 20°. The phase profiles of the metasurfaces were obtained by imposing appropriate constraints on the rays’ OGLs (Supplementary Information) and are presented in Supplementary Fig. S5. Simulated intensity distributions for each design are presented in Supplementary Fig. S6. A single-layer metasurface deflector was also designed to serve as a control.

Figure 4b shows the materials and geometry used to implement the beam deflectors. Instead of placing the metasurfaces on opposite sides of a substrate, we employed a folded metasystem architecture^27^ (Fig. 4b, left): light deflected by the first metasurface propagates to the opposite side of the substrate and is reflected towards the second metasurface by a gold mirror. This layout allows both metasurfaces to be located on the same side of the substrate, simplifying fabrication. The metasurfaces were implemented using a high-contrast transmitarray platform^21^. Each metasurface is composed of an array of amorphous silicon nano-posts with square cross-sections and equal heights (Fig. 4b). The nano-posts rest on a fused silica substrate and are encapsulated from above with SU-8 polymer. Curves relating the transmission phase to the post width are presented in Supplementary Fig. S7; see ‘Materials and methods’ for the details of the metasurface design.

The beam deflectors were fabricated using standard nanofabrication tools and techniques. We first deposited amorphous silicon on a fused silica substrate. The nano-post patterns were defined in a layer of resist using electron beam lithography and transferred to the amorphous silicon layer via a plasma etching process. The protective SU-8 layer was spun before the deposition of gold on the opposite side of the substrate. Optical and scanning electron images of the fabricated devices are shown in Fig. 4c.

**Fig. 5:**
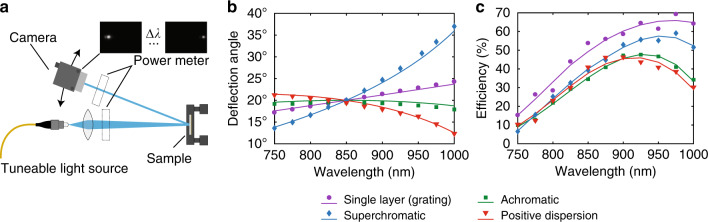
Beam deflector characterization. **a** Schematic of the measurement setup. The sample is illuminated by a tuneable light source, and the deflected beam is imaged on a camera. **b** Deflection angles for the control, achromatic, positive-dispersion, and superchromatic beam deflectors as functions of wavelength. The solid curves represent the simulated responses. **c** Deflection efficiencies of the beam deflectors as functions of wavelength. The solid lines serve as guides for the eye. The power measurements were obtained at the locations indicated in **a**.

To characterize the dispersion, we illuminated the input metasurface of each beam deflector with an unpolarized tuneable light source and imaged the deflected beams on a camera. A schematic depicting the measurement setup is shown in Fig. 5a. Representative spectral data for the tuneable light source are shown in Supplementary Fig. S8. The measured deflection angles for light with wavelengths between 750 and 1000 nm in steps of 25 nm are shown in Fig. 5b along with the expected angles found through simulation (see ‘Materials and methods’). The measured deflection angles for all devices show good agreement with the simulated angles.

The measured deflection efficiencies, where this efficiency is defined as the ratio of the power of the deflected beam to that of the incident beam, are shown in Fig. 5c. All devices show an efficiency peak between 900 and 975 nm. These peaks are red-shifted and show reduced efficiency compared with what modelling predicts (Supplementary Figs. S9 and S10). This discrepancy is likely the result of fabrication imperfections. Compared to a single-surface deflector, a cascaded system is expected to show reduced transmission efficiency, as the losses at each individual surface accumulate. The metasurface design could be further optimized to achieve higher efficiency^36^.

## Discussion

In a system comprising many metasurfaces, light of different wavelengths can propagate along significantly different paths. In this article, we derive a relation connecting the paths through an optical system along which light of a specific wavelength travels to the system’s chromatic behaviour. This relation allows us to concisely state the achromaticity criterion for focusing systems: in an achromatic system, the OGLs of all ray paths are equal. We propose a design framework for achromatic optics based on predefined trajectories and demonstrate its application with two examples. We extend this design framework to systems with arbitrary dispersion and validate it experimentally by demonstrating superchromatic and achromatic beam deflectors and a beam deflector exhibiting positive dispersion.

The simplicity of the OGL design paradigm facilitates the discovery of unconventional optical components and systems. Although all metasystems discussed in this work comprise only two diffractive elements separated by a planar substrate, our design framework is applicable to systems composed of arbitrarily many refractive or diffractive elements, including refractive–diffractive hybrids. Systems of three or more metasurfaces may provide sufficiently many degrees of freedom to account for higher-order dispersion terms^12^ or correct off-axis aberrations^26^, thus enabling a new class of optical systems that can achieve any desired chromatic response.

## Materials and methods

### Simulation results

The phase profiles for the achromatic beam deflector (Fig. 2b), achromatic metalens (Fig. 3b), apochromatic metalens (Supplementary Fig. S3b), and fabricated beam deflectors (Supplementary Fig. S5) were obtained by first determining the ray paths necessary to achieve the desired dispersion (see the Supplementary Information) and then numerically computing the phase surfaces to direct rays along these paths. As shown in the Supplementary Information, two solutions exist for each prescribed dispersion: one branch was chosen for the achromatic and positive beam deflectors, and the other was chosen for the superchromatic beam deflector. This is illustrated in the ray diagrams in Supplementary Fig. S5, in which rays travel from left to right (right to left) for the achromatic and positive-dispersion (superchromatic) beam deflector(s). The choice of solution is also distinguished by the sign of the phase gradient for the first metasurface, ${\mathrm d}\phi _1/{\mathrm d}x$. The solutions were chosen on the basis of the second-order dispersion coefficients.

The simulated optical intensity distributions presented in Figs. 2c, 3d, e and Supplementary Figs. S1–4 and the simulated intensity distributions for the fabricated beam deflectors that are shown in Supplementary Fig. S6 were obtained by modelling the metasurfaces as ideal phase masks that do not modify the local optical intensity. The propagation of the optical waves in the regions before, between, and after the metasurfaces was performed using the plane wave expansion technique, which is a full-wave vectorial approach that does not involve any approximations^2^. The incident waves in Fig. 2c and Supplementary Fig. S6 were normally incident Gaussian beams with a waist radius of 130 μm whose waist was in the plane of the first metasurface. The incident waves for the results shown in Fig. 3d, e and Supplementary Figs. S1–3 were normally incident plane waves; for the results in Supplementary Fig. S4, plane waves with the indicated incidence angles were used. The simulated deflection angles shown in Figs. 2d and 5b correspond to the angles of the peak intensity in the far-field patterns of the deflected beams. The variations in the focal lengths with the wavelength that are presented in Fig. 3c for the twisted metalens and the annular single-layer metalens show the corresponding displacements of the peaks of the simulated intensity distributions presented in Fig. 3d, e and Supplementary Fig. S2 as functions of wavelength.

### Metasurface design

To design the meta-atoms for these metasurfaces, we first obtained the complex transmission amplitude *t*(*w*) as a function of the post width *w*, which was determined using a rigorous coupled-wave analysis solver^37^. We chose a lattice constant of *a* = 300 nm, which satisfies the Nyquist sampling criterion $a < \lambda /\left( {n_1 + n_2} \right)$ (ref. ^38^) for arbitrary incidence and deflection angles for refractive indices of $n_1 = 1.45$ and $n_2 = 1$ and at all wavelengths *λ* within the range considered. The amplitude and phase of *t*(*w*) as functions of the post width are shown in Supplementary Fig. S7a. The optimal post width $w(\varphi )$ for a given transmission phase *φ* was chosen by maximizing ${\mathrm{Re}}\{ t(w){\mathrm e}^{i\varphi }\}$. These optimal widths are shown as a function of the phase in Supplementary Fig. S7b. In these simulations, we used refractive indices of *n* = 1.45, 1.56, and 3.84 for fused silica, SU-8, and α-Si, respectively. The input metasurface of each beam deflector had dimensions of 500 μm × 500 μm; the dimensions of the output metasurface were selected to fully contain the output beam for a wavelength range of 700–1000 nm and were obtained from the simulations presented in Supplementary Fig. S6. The output metasurface dimensions for the beam deflectors were also 500 μm × 500 μm.

### Device fabrication

First, a 485-nm-thick layer of amorphous silicon (α-Si) was deposited on one side of a 1-mm-thick fused silica substrate using plasma-enhanced chemical vapour deposition. An approximately 200-nm-thick layer of positive electron beam resist (ZEP 520A-7, Zeon Chemicals) was spin-coated on top of the α-Si layer, followed by a 70-nm-thick layer of conductive polymer (AR-PC 5090, Allresist), which was applied to mitigate charging during electron beam lithography. The beam deflector patterns were exposed using a 125 keV electron beam lithography system (ELS-F125, Elionix). The water-soluble conductive polymer was removed, and the resist was developed in a developer (ZED-N50, Zeon Chemicals). A hard mask was created by first evaporating approximately 50 nm of aluminium oxide onto the resist and then lifting it off in a solvent. The pattern was then transferred to the α-Si layer by means of plasma etching in C_4_F_8_ and SF_6_ chemistry. The hard aluminium oxide mask was removed in a mixture of ammonium hydroxide and hydrogen peroxide heated to 80 °C. Figure 4c shows a scanning electron micrograph of the sample at this stage. The nano-posts were encapsulated in an approximately 5-μm-thick layer of SU-8 photoresist. The SU-8 was spin-coated, baked at 95 °C to reflow, exposed, and cured to form a permanent protective layer over the nano-posts. On the opposite side of the substrate, a similar layer of SU-8 was deposited to act as an adhesion layer. We then evaporated approximately 50 nm of gold onto this surface to form the folding mirror.

### Measurement procedure

Our measurement setup is shown schematically in Fig. 5a. Our tuneable light source comprised a supercontinuum light source (YSL SC-5-FC) and a monochromator (Acton Research Corporation SpectraPro 2150i) coupled to a single-mode fibre. The source exhibited a full-width at half-maximum of approximately 0.7 nm in the 700–1000 nm spectral range. Normalized spectra at representative wavelengths are shown in Supplementary Fig. S8. The light exiting the fibre was gently focused to produce a spot size of approximately 150 μm at the sample, which was located approximately 25 cm from the lens. The beam deflector sample was affixed to a mirror mount attached to a three-axis micropositioning stage. This allowed us to position the incident beam to impinge on a specific metasurface at normal incidence. A camera (Edmund Optics EO-5012M) was mounted on a rotation stage with the rotation axis centred on the sample. As the deflected beam approached the limits of the image sensor, a reference image was taken, and the camera was repositioned, allowing image fields acquired at different positions to be stitched together. The deflection angles were determined by calculating the intensity centre of mass for these images. Sample images are shown in Supplementary Fig. S11.

The same setup was used to measure the deflection efficiency. A calibrated power meter (PM100D with an S120C head, Thorlabs) was placed at the positions indicated in Fig. 5a. The power meter was moved to follow the deflected beam as the wavelength of the incident light was tuned. The efficiencies reported in Fig. 5c are the deflected-to-incident power ratios. The reflectivity at near-normal incidence of the unpatterned region was measured to be 94 ± 2% across the 700–1000 nm band. This is predominantly due to the gold mirror but also includes reflection from the air–SU-8 interface.

### Deflection efficiency simulation

To assess the performance of the beam deflectors, we first performed finite-difference time-domain simulations^39^ of the single-surface beam deflector. An illustration of the simulation geometry and representative cross-sectional fields are shown in Supplementary Fig. S9. In each simulation, the structure was excited by a normally incident monochromatic plane wave originating in the SU-8. The fields at the output planes were projected onto ideal fields, and the efficiency was calculated by dividing the power in each projected field by the incident power. Both *x*- and *y*-polarized incident fields were simulated. The simulated efficiencies are shown alongside the measured data in Supplementary Fig. S10.

## Supplementary information

Supplementary Information

## Data Availability

The main data supporting the findings of this study are available within the article and its Supplementary Information. Additional data are available from the corresponding author upon reasonable request.
