# Supplementary material for: At-will chromatic dispersion by prescribing light trajectories with cascaded metasurfaces
Source: Light Sci Appl. 2020 May 27;9:93. doi: 10.1038/s41377-020-0335-7 (PMC7253474; doi:10.1038/s41377-020-0335-7)
Supplement: Supplementary file 1 — Supplementary Information [file 41377_2020_335_MOESM1_ESM.pdf]

Supplementary information for

At-will chromatic dispersion by prescribing light trajectories with cascaded metasurfaces

Andrew McClung,[1] Mahdad Mansouree,[1] and Amir Arbabi[1]

[1]*Department of Electrical and Computer Engineering, University of Massachusetts Amherst, 151 Holdsworth Way, Amherst, MA 01003, USA*

SUPPLEMENTARY FIGURES

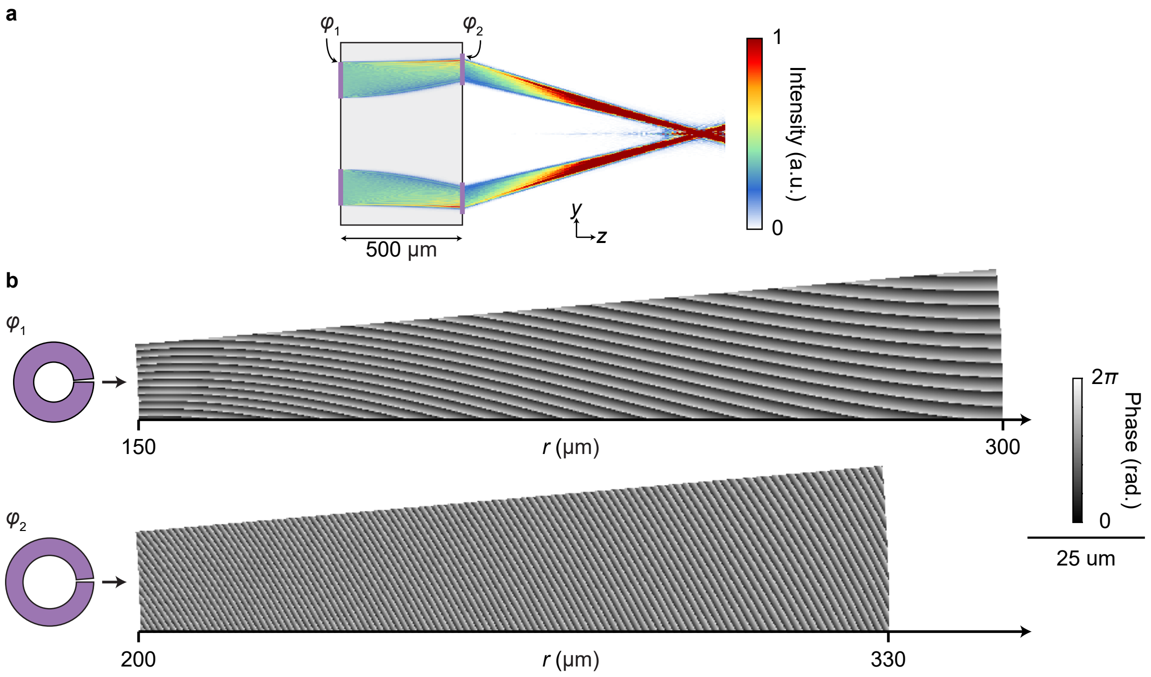

Figure S1 | Cross-sectional intensity and phase profiles for the annular achromatic doublet. (a) Transverse intensity distribution for light focused by the bilayer metalens presented in Fig. 3d, and **(b)** segments of the phase masks for each metasurface.

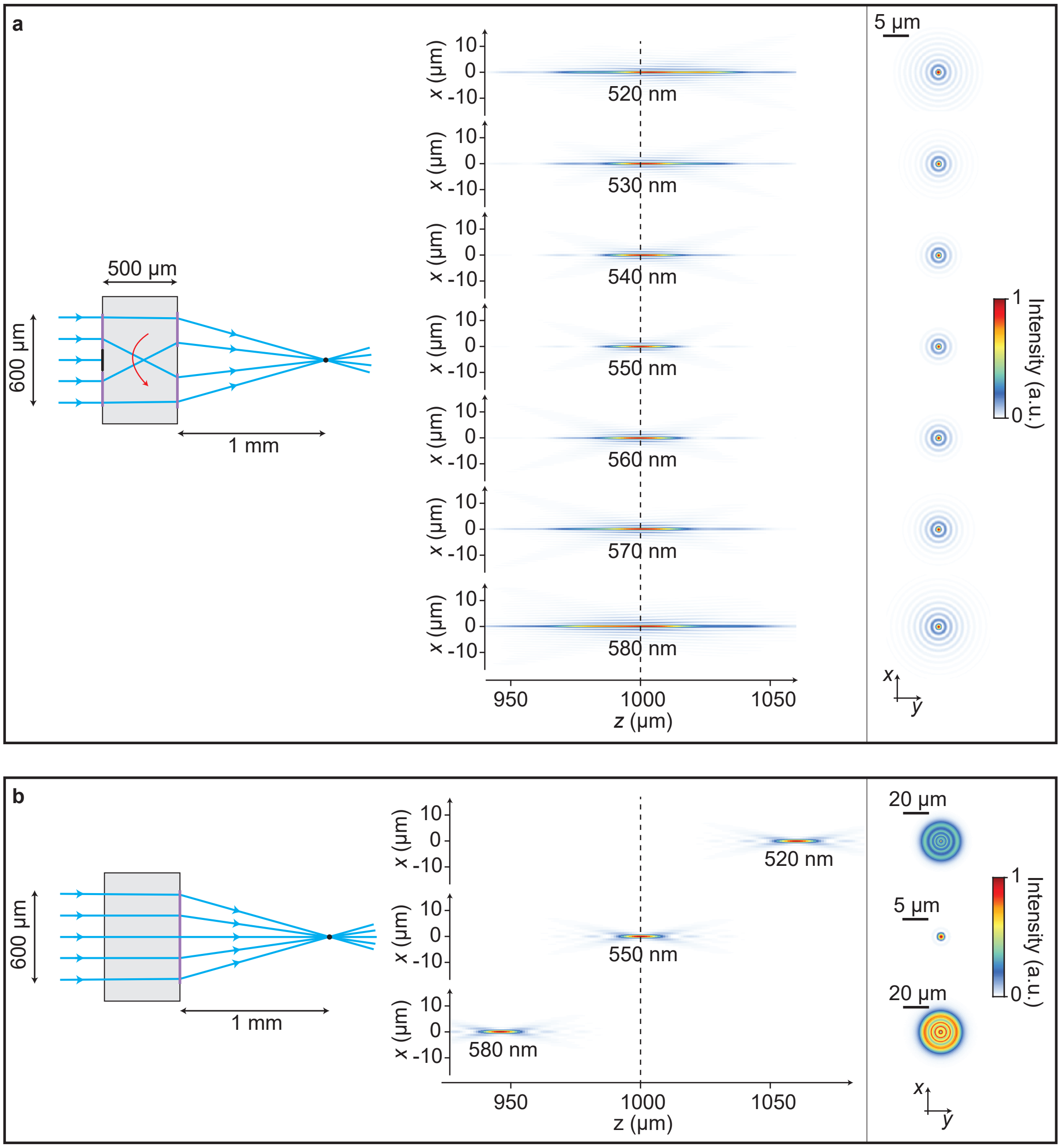

Figure S2 | Intensity distributions for annular achromatic doublet and single-layer circular aperture metalens. **(a)** Axial and transverse intensity distributions for the bilayer metalens presented in Fig. 3d. **(b)** Similar intensity distributions for a single-layer metalens with circular aperture.

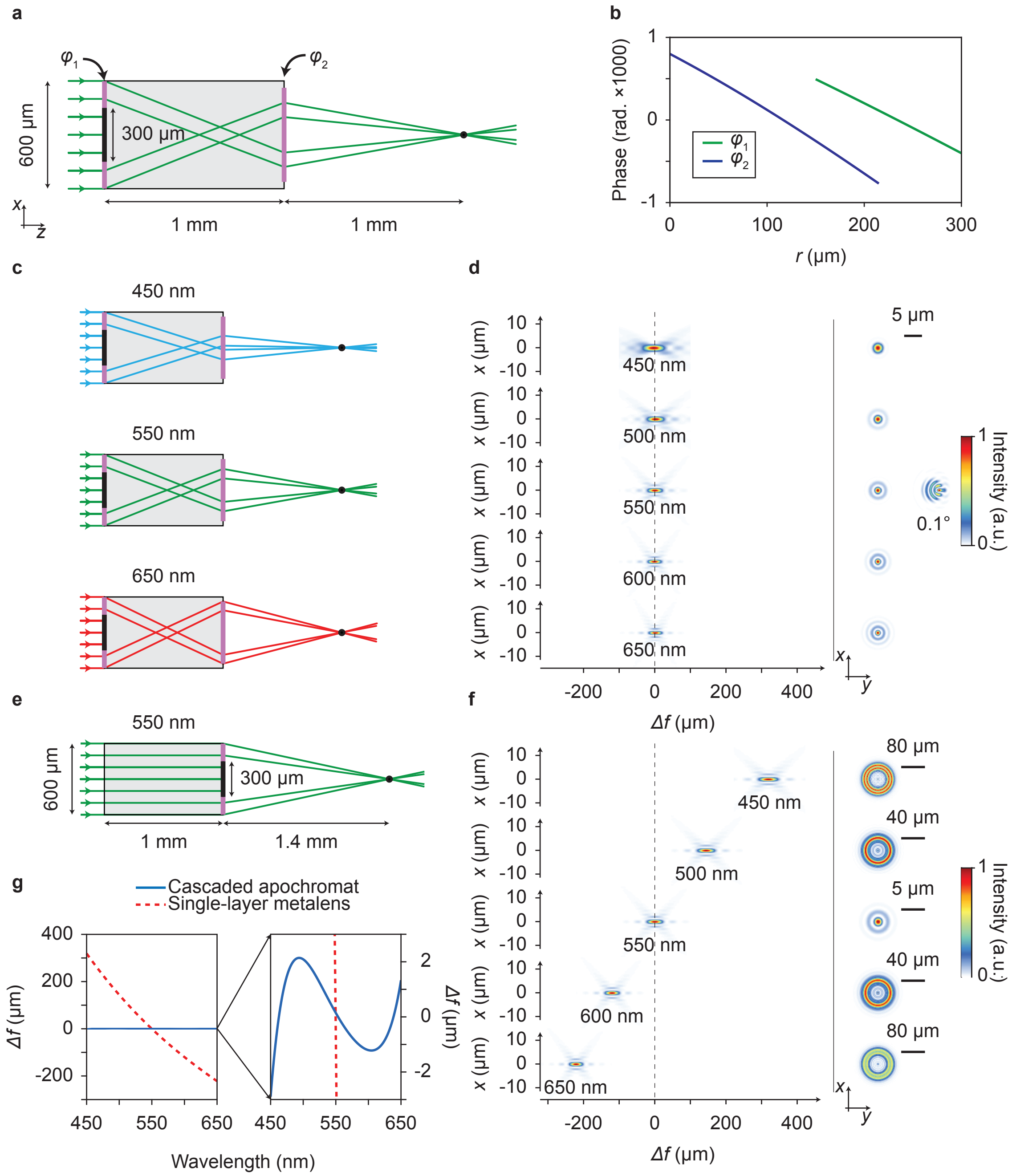

Figure S3 | Apochromatic doublet. **(a)** Schematic and **(b)** radial phase profiles of bilayer apochromat. **(c)** Ray diagrams for light incident at 450 nm, 550 nm and 650 nm. **(d)** Axial and transverse ($\Delta f = 0$) intensity distributions for apochromat. The transverse intensity profile for 550 nm light with 0.1° incidence is also shown. **(e)** Ray diagram for a single-layer metalens with same NA as the apochromat at 550 nm. **(f)** Axial and transverse intensity distributions for single-layer metalens. **(g)** Wavelength dependence of the focal lengths of apochromat and single-layer metalens. Inset shows magnified view.

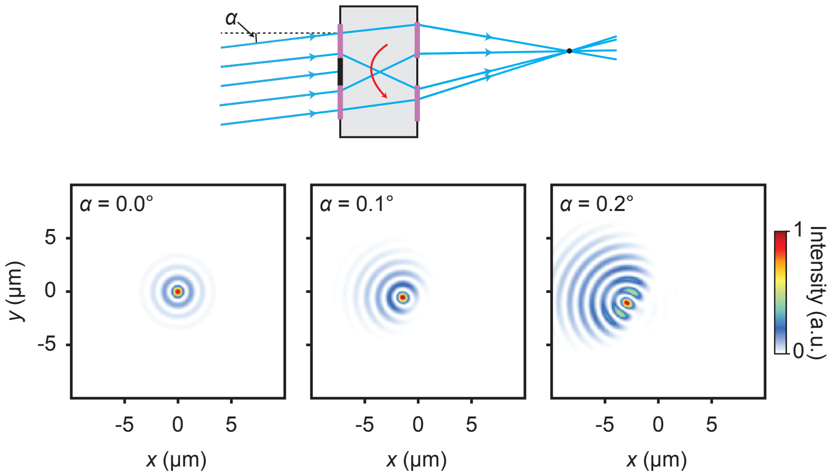

Figure S4 | Transverse intensity distribution for annular achromatic doublet. Intensity distributions for light at 550 nm at normal incidence (left), 0.1° (centre) and 0.2° (right).

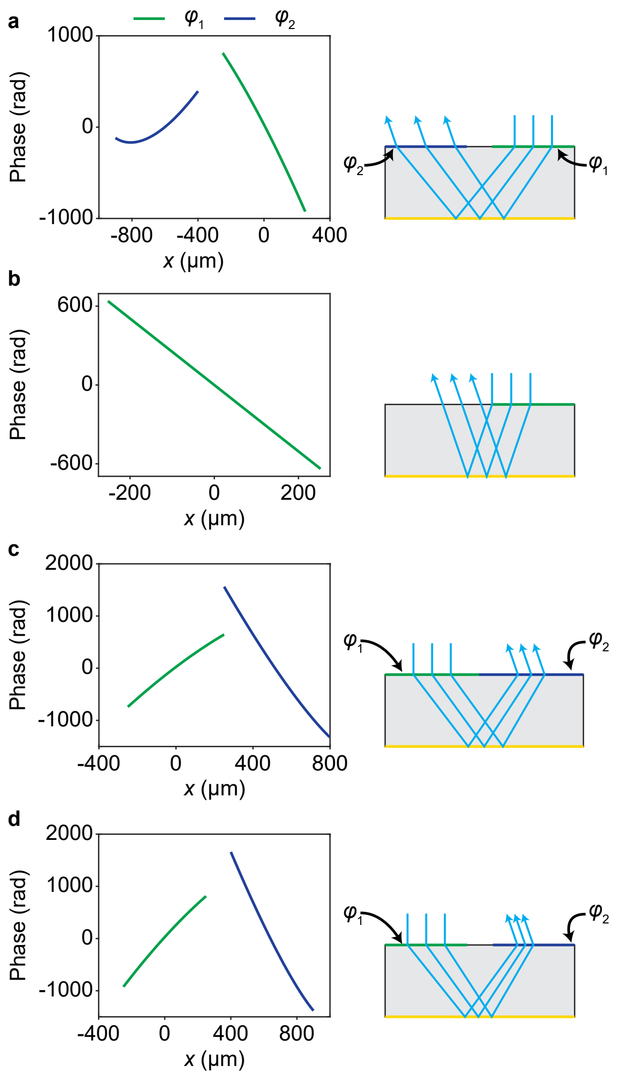

Figure S5 | Beam deflector phase profiles. Metasurface phase profiles for **(a)** superchromatic, **(b)** ordinary grating, **(c)** achromatic, and **(d)** positive dispersion beam deflectors. In each panel the green curve corresponds to the profile for the input metasurface (ϕ_1), and in panels a, c, and d, the blue curve corresponds to the profile for the output metasurface (ϕ_2). Diagrams to the right of each graph depict ray paths (not to scale).

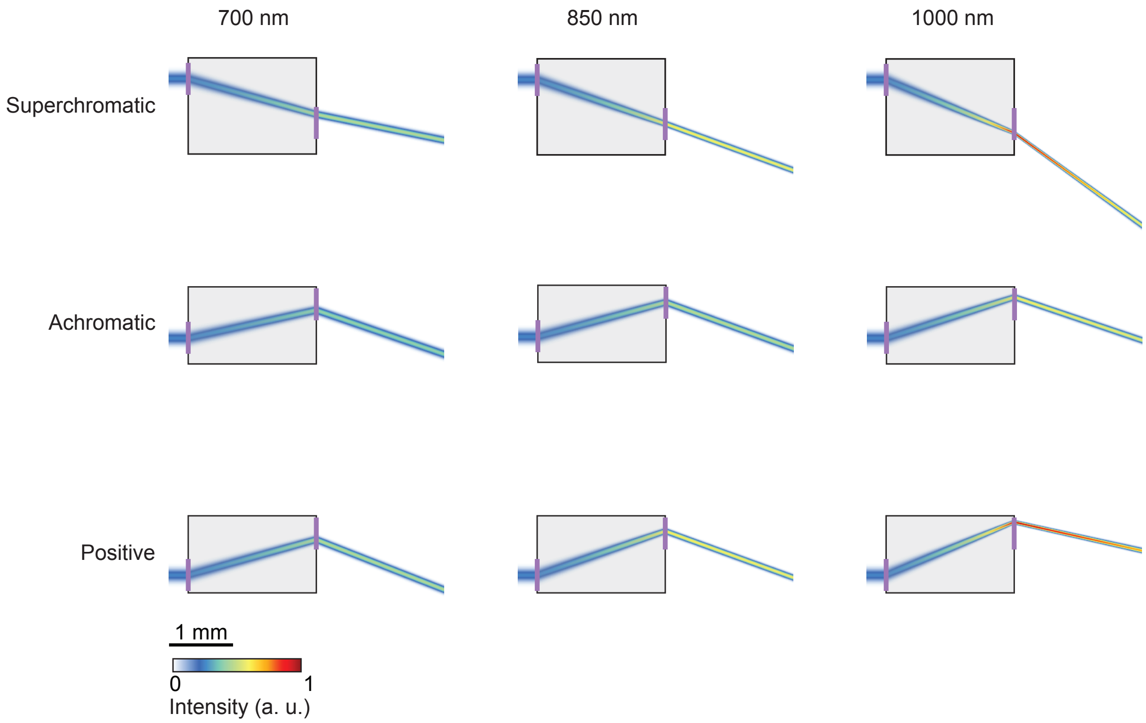

Figure S6 | Simulated beam deflector intensity distributions. Metasurfaces are specified as ideal phase masks. Intensity distributions are obtained by Fourier propagation of a Gaussian beam (beam waist: $w = 130$ μm).

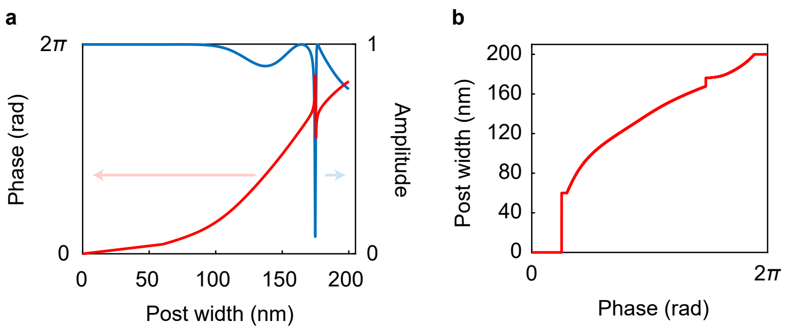

Figure S7 | Metasurface design curves. (a) Absolute value (blue) and phase (red) of transmission amplitude as a function of post width. **(b)** Optimized post width as a function of phase.

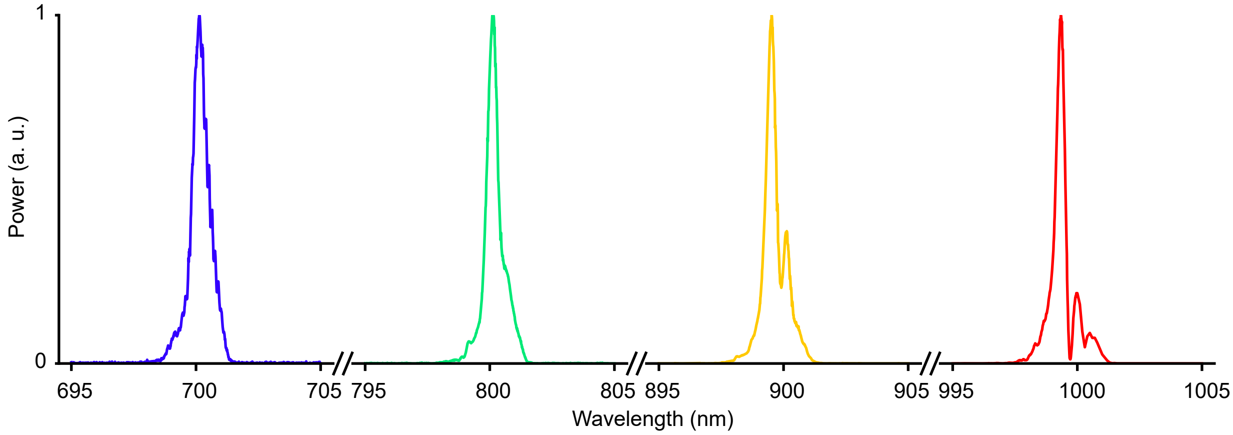

Figure S8 | Optical spectra of tuneable light source. Curves show spectra for four different representative monochromator settings (700 nm, 800 nm, 900 nm and 1000 nm). Each dataset is normalized to its peak power.

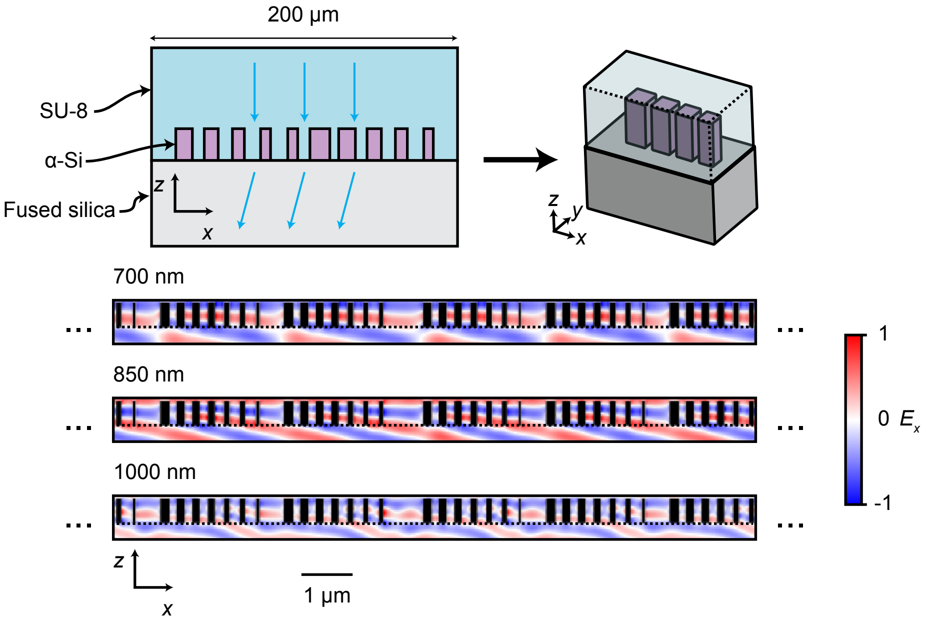

Figure S9 | Grating deflection efficiency: FDTD simulation. Deflection efficiency for a 200-µm-wide, 20 degree beam deflector as a function of wavelength is determined by 3D FDTD simulations. For each wavelength, a monochromatic plane wave originating in the SU-8 layer is directed towards the nano-posts and an output plane below the nano-posts is recorded.

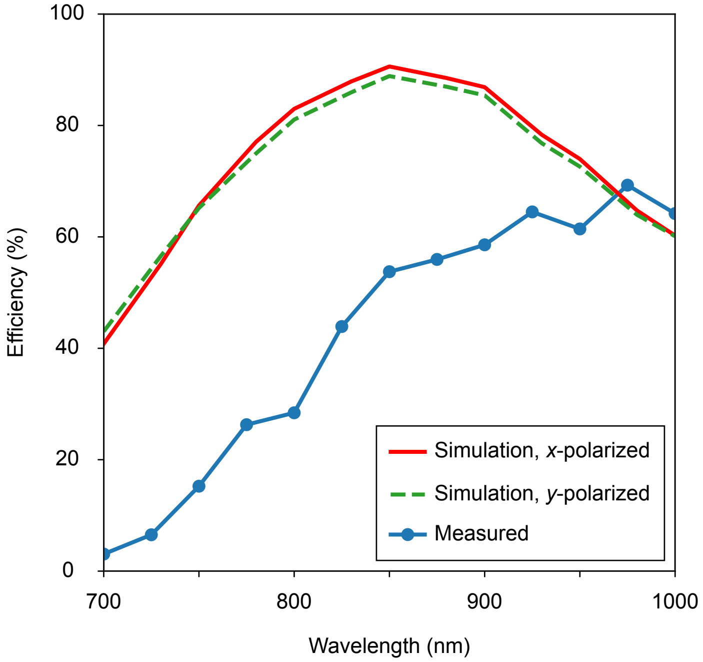

Figure S10 | Grating deflection efficiency: simulation vs. measurement. Results of FDTD simulation for x-polarized (red, solid) and y-polarized (green, dashed) incident fields alongside measured efficiencies (blue, circles).

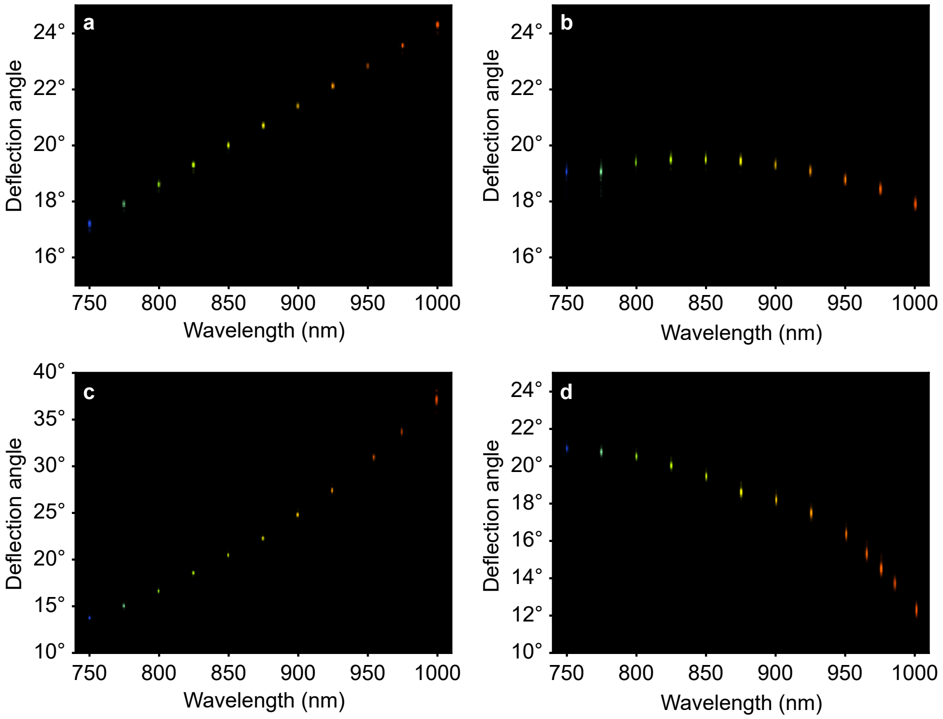

Figure S11 | Images of deflected beams. Imaged beams for the **(a)** grating, **(b)** achromatic, **(c)** superchromatic, and **(d)** positive dispersion beam deflectors as a function of wavelength.

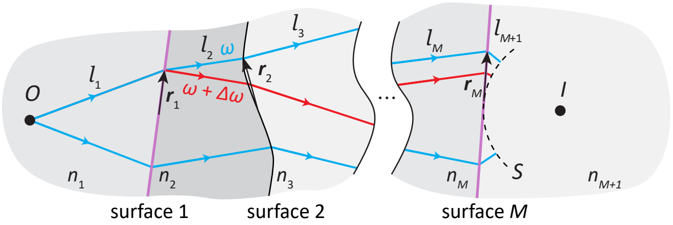

Figure S12 | Illustration of focusing by a cascaded metasurface system (extended). This figure depicts the same system as Fig. 1 but adds additional detail. Vectors $\mathbf{r}_1, \ldots \mathbf{r}_M$ indicate the intersection of the fiducial ray with surfaces $1 \ldots M$. The dashed line indicates a reference sphere S centred at I.

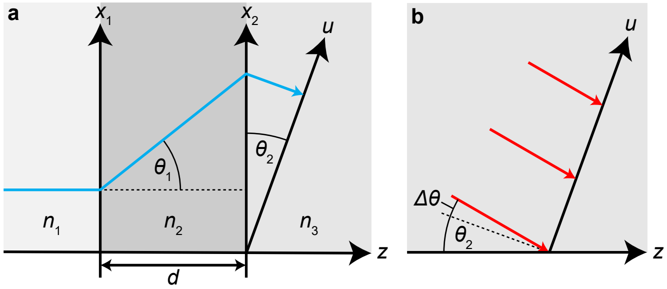

Figure S13 | Engineering dispersion. (a) Diagram of a ray traversing the beam deflector system, defining coordinate axes x_1, x_2, u, and z, deflection angles θ_1 and θ_2 and indicating regions with refractive indices n_1, n_2, and n_3. **(b)** Diagram showing a plane wave arriving at u with oblique incidence.

SUPPLEMENTARY TEXT

Achromaticity criterion. Consider the optical system depicted in Fig. S12. The path of a ray through the system at its design frequency ω can be parameterized by a set of position vectors $\{\mathbf{r}_1, \mathbf{r}_2, \ldots, \mathbf{r}_M\}$, where $\mathbf{r}_m$ represents the intersection of the path with the mth surface. We designate as fiducial the ray which impinges the first surface at $\mathbf{r}_1$. If frequency is changed to $\omega + \Delta\omega$, the deflection of the fiducial ray at each interface is modified, altering its trajectory: its intersection with the mth surface becomes $\mathbf{r}_m + \Delta\mathbf{r}_m$, where $\Delta\mathbf{r}_m = \mathrm{d}\mathbf{r}_m/\mathrm{d}\omega\,\Delta\omega$. At frequencies other than ω, the fiducial ray still emanates from O but does not necessarily pass through I, so we define l_{M+1} as the distance the ray travels between the last surface and a reference sphere [1] centred at I with radius equal to the minimum distance between I and the Mth surface (see Fig. S12). To first order in $\Delta\omega$, the accumulated phase changes by

$$\Delta\Phi = \left(\frac{\partial\Phi}{\partial\omega} + \sum_{m=1}^{M} \nabla_m\Phi \cdot \frac{\mathrm{d}\mathbf{r}_m}{\mathrm{d}\omega}\right)\Delta\omega, \tag{S1}$$

where ∇_m represents the gradient with respect to $\mathbf{r}_m$. According to Fermat's principle, the total phase acquired is stationary with respect to path variations ($\nabla_m\Phi = 0$ for all m) [1], so the second term in parentheses vanishes, leaving $\Delta\Phi = (\partial\Phi/\partial\omega)\,\Delta\omega$. Though path variation has no effect on $\Delta\Phi$ to first order, it does contribute at second and higher orders, which determine the bandwidth of an achromatic system.

The system focuses rays to point I at frequency $\omega + \Delta\omega$ if and only if the phase accumulated along each ray's path ($\Phi + \Delta\Phi$) is the same. Because Φ is the same for each ray, this implies the system is achromatic if and only if $\Delta\Phi$ or equivalently l_g is the same for all rays. Equation 2 is obtained by partial differentiating Eq. 1 and multiplying by c.

Design of bilayer apochromat. We present a design for a bilayer apochromatic metalens—a lens with the same focal length at three wavelengths—in Fig. S3. Like the twisted metalens presented in the main text, the apochromat has an annular aperture (150 µm< r_1 <300 µm) and 1 mm focal length, as shown schematically in Fig. S3a. Unlike the twisted metalens, the apochromat achieves the OGL criterion at two wavelengths and only deflects rays radially. The metalens comprises two metasurfaces that are separated by a 1-mm-thick substrate (n=1.46); their radially symmetric phase profiles for are shown in Fig. S3b.

The apochromat is designed to work at visible wavelengths, and is well-corrected over the 450–

650 nm range. The metalens's effective NA increases with wavelength across this band, as can be seen in the ray diagrams in Fig. S3c: shorter wavelengths (450 nm) are deflected to a shallow angle, arriving at the second metasurface closer to its centre, whereas longer wavelengths (650 nm) are deflected to a steeper angle by the first metasurface, and arrive at the second metasurface farther from the optical axis. In the centre of its operating bandwidth (550 nm), the apochromat has an effective NA of 0.21. The consequences of the wavelength-dependent NA are apparent in the transverse and axial intensity profiles (Fig. S3d). The apochromat, like the twisted metalens, has a narrow field of view, as evidenced by the transverse intensity profile for a 550 nm field incident at 0.1°.

To benchmark the performance of the bilayer apochromatic metalens, we designed a single-layer hyperbolic metalens with the same annular aperture, shown schematically in Fig. S3e. This control lens was designed to have NA=0.21 at its design wavelength of 550 nm, matching that of the apochromat and producing a focal length of 1.4 mm. The transverse and and axial intensity profiles are shown in Fig. S3f, demonstrating chromatic behaviour qualitatively similar to the other single-layer metalenses presented in this work (see Fig. 3e and Fig. S2b).

The shift of focal length with wavelength of these two metalenses are compared in Fig. S3g. The single-layer metalens exhibits a shift in focal length of Δf ≈500 µm over the band considered, consistent with the expected behaviour for a non-dispersive, single-layer metalens with this focal length [2]:

$$\frac{\Delta f}{f} = \frac{\Delta \lambda}{\lambda}. \tag{S2}$$

By contrast, the focal length of the apochromat shifts less than 5 µm over the same range.

Designing beam deflector dispersion. Figure S13a shows the path of a ray through a system of two metasurfaces. Assume the ray is at the design frequency ω. The first metasurface lies on the x_1 axis, and deflects the normally-incident ray to an angle θ_1. The second metasurface lies on the x_2 axis, parallel to and a distance d from x_1, and deflects the ray to an angle θ_2. The deflection angle for the beam deflector system is θ_2, and thus is the same for all rays at frequency ω; θ_1 may vary among rays. The u axis makes an angle θ_2 with the x_2 axis, and the origin for each axis lies at their intersection. The medium between x axes has refractive index n_2 and the medium to the right of x_2 has refractive index n_3. At the design frequency, the total phase accumulated from x_1 to u is constant for all rays. The group length for the ray can be expressed in these coordinates and

media as

$$l_{\mathrm{g}}(u) = \frac{n_2 d}{\cos\theta_1} + n_3 u \tan\theta_2. \tag{S3}$$

Note that for fixed u and θ_2 there are two values of $\theta_1 \in [-\pi, \pi)$ that satisfy Eq. S3.

We will impose the requirement that $l_{\mathrm{g}}(u) = au + l_0$. We can then write the phase change along u due to a frequency change $\Delta\omega$ as

$$\Delta\Phi(u) = \frac{\Delta\omega}{c}\left(au + l_0\right). \tag{S4}$$

Compare this with the phase profile for a plane wave arriving at u at oblique incidence (Fig. S13b),

$$\Delta\Phi(u) = \Phi_0 - \frac{n_3\omega}{c} u \sin\Delta\theta \tag{S5}$$

where $\Delta\theta$ is the angle of the wavefront with respect to u. Equating first-order coefficients in Eq. S4 and Eq. S5 gives

$$\frac{\Delta\theta}{\Delta\omega} = -\frac{a}{n_3\omega}, \tag{S6}$$

where we have used $\sin\Delta\theta \approx \Delta\theta$. Choosing $a = \tan\theta_2$ gives ordinary grating dispersion, and $a = 0$ gives the achromatic condition. Superchromatic and positive behaviour results from exceeding these bounds.

For a given value of a and a choice of l_0 we can solve Eq. S3 for $\theta_1(u)$. Coordinates x_1, x_2 and u are related by

$$x_2 = \frac{u}{\cos\theta_2} \tag{S7}$$

and

$$x_1 = x_2 - d\tan\theta_1. \tag{S8}$$

Phase surfaces are then designed to produce the necessary deflections at x_1 and x_2.

[1] M. Born and E. Wolf, *Principles of Optics: Electromagnetic Theory of Propagation, Interference and Diffraction of Light*, 7th ed. (Cambridge University Press, Cambridge, 1999).

[2] E. Arbabi, A. Arbabi, S. M. Kamali, Y. Horie, and A. Faraon, Multiwavelength polarization-insensitive lenses based on dielectric metasurfaces with meta-molecules, Optica **3**, 628 (2016).
